# Controlled release of silica-coated insulin-loaded chitosan nanoparticles as a promising oral administration system

**DOI:** 10.1186/s40360-023-00662-1

**Published:** 2023-03-30

**Authors:** Mohamed M. Fathy, Asmaa A. Hassan, Anwar A. Elsayed, Heba M. Fahmy

**Affiliations:** Biophysics Department, Faculty of Science, Cairo University, Giza, Egypt

**Keywords:** Oral administration, Silica coat, Insulin-loaded chitosan nanoparticles, Drug release test

## Abstract

**Background:**

Oral insulin administration has recently become one of the most exciting research subjects. Different approaches have been carried out to get an effective oral insulin delivery system using nanotechnology. The development of a delivery system that overcomes the difficulties of oral insulin administration, achieving high stability and minimal side effects, is still an urgent need. Therefore, this study is considered one of the efforts to design a new prospective drug delivery nano-composite (silica-coated chitosan-dextran sulfate nanoparticles).

**Methods:**

Chitosan-dextran sulfate nanoparticles (CS-DS NPs) were prepared via a complex coacervation method and then coated with silica. Uncoated and silica-coated CS-DS NPs were physically characterized via different techniques. Transmission electron microscopy (TEM), scanning electron microscopy (SEM), energy-dispersive X-ray (EDX) analysis, and atomic force microscopy (AFM) have been used to investigate the chemical elements, size, morphology, and surface properties of the prepared formulations. Differential scanning calorimetry (DSC) to assess the thermal properties of formed nano-formulations. Fourier transform infrared (FT-IR) spectroscopy investigated the silica coat and chitosan interaction. The encapsulation efficiency was evaluated using high-performance liquid chromatography (HPLC) analysis. The insulin release profile of nano-formulations was performed with and without silica coat at two different pHs (5.5,7), nearly simulating the environment of the gastrointestinal tract (GIT).

**Results:**

The silica-coated CS-DS NPs revealed interesting physicochemical properties exemplified by suitable core particle size obtained by TEM images (145.31 ± 33.15 nm), hydrodynamic diameter (210 ± 21 nm), high stability indicated by their zeta potential value (-32 ± 3.2 mV), and adequate surface roughness assessed by AFM. The encapsulation efficiency of insulin-loaded chitosan nanoparticles (ICN) was (66.5%) higher than that of insulin-chitosan complex nanoparticles (ICCN). The silica-coated ICN demonstrated a controlled insulin release profile at pHs (5.5 and 7) compared with uncoated ICN.

**Conclusion:**

The silica-coated ICN can be an efficient candidate as a desired oral delivery system, overcoming the common obstacles of peptides and proteins delivery and achieving high stability and controlled release for further applications.

## Background

One of the most significant nano-medical applications is designing a smart drug delivery nano-carrier. The administration of drugs orally has been considered the most preferable over other routes of drug delivery regarding the pain and possible infections caused by them [1, 2]. However, the oral bioavailability of some therapeutic agents is limited due to incomplete absorption, the influence of pH alteration through GIT on the drug and drug carriers, and the enzymatic degradation of many medications, particularly peptides and proteins, leading to their poor stability [3, 4].

Several approaches have been developed to overcome these problems and enhance the therapeutic effect of orally administrated protein and peptide pharmaceuticals like insulin [5]. Recently, oral insulin administration for managing type 2 diabetes developed as an alternative method to the traditional delivery of insulin through injection [6].

For oral insulin administration, many drug nano-carrier have been developed to protect insulin from biodegradation and enhance transmucosal absorption [7, 8]. Biodegradable polymeric nano-carrier, such as the cationic polysaccharide chitosan (CS), was introduced to beat some difficulties of drug stability in GIT and control the drug release due to its favorable properties, such as much-adhesiveness, permeation-enhancing capability, biocompatibility, and low toxicity [9, 10]. Mukhopadhyay et al. reported the self-assembly of chitosan/insulin nanoparticles for oral insulin delivery. The prepared nano-formulation average size range was about 200—550 nm with encapsulation of about 85% [11].

However, chitosan nano-carriers encounter some obstacles, such as their high solubility at low pH, causing earlier leakage of insulin within the stomach [12]. To overcome this problem, chitosan nano-composites have been developed using the ionic gelation method with an oppositely charged ionic polymer such as dextran sulfate (DS) to provide a controlled released and stable drug delivery system due to better crosslinking of CS with DS during the formation of nano-carrier [13]. Pechenkin et al. formed chitosan-dextran sulfate nano-formulation for oral insulin delivery [14]. This preparation achieved a relatively high encapsulation efficiency (65%), and the in vitro release was studied at different pH values simulating the GIT tract. Additionally, mesoporous silica nanoparticles coated with Chitosan have been designed as an injectable insulin delivery system. The interaction of insulin with CS and silica was indicated by surface tension studies showing high encapsulation efficiency, which reflects favorable obtained results [15].

Accordingly, administering insulin orally is considered a significant challenge in drug delivery. Therefore, this study aims to improve the release properties of CS-DS NPs using a biocompatible and non-toxic silica coat. The modified Stober method achieved the coating technique [16] to enhance the pH stability of CS-DS NPs and increase their therapeutic effect.

## Materials and methods

### Materials

Human insulin (100 IU/ml) was brought from VACSERA, Egypt. Chitosan (medium molecular weight, degree of deacetylation 96%), tetraethyl orthosilicate (TEOS), and sodium hydroxide (NaOH) were obtained from Sigma Aldrich, Germany. Dextran sulfate sodium salt was provided from Applichem, Germany. Acetic acid (99.7%) and Ammonium hydroxide solution (NH_4_OH) were obtained from Piochem for Egypt's laboratory chemicals. Ethanol (96%) was purchased from Diachem chemicals. Hydrochloric acid (HCL) was obtained from SDFCL, India.

## Methods of preparation

### Preparation of Chitosan- dextran sulfate nanoparticles (CS-DS NPs)

CS-DS nano-carriers were formed using a complex coacervation method [17]. Chitosan (0.1 g) was dissolved in 100 ml glacial acetic acid solution (1%) and was left on a magnetic stirrer overnight at room temperature. DS solution was prepared by dissolving 0.1 g DS in 100 ml deionized water. Then, 50 ml of DS solution was added dropwise to 75 ml of CS solution under stirring, so the ratio of CS to DS becomes 1.5:1. After nanoparticle formation, the solution was centrifuged at a temperature of 25 °C and 10,000 rpm for 20 min to collect the formed CS-DS NPs that were washed using deionized water three times to remove any chemical residuals.

### Encapsulation of insulin

Human insulin (100 IU/ml) was encapsulated to the prepared CS-DS NPs at two different pHs, which consequently affects the encapsulation efficiency of insulin.

### (A) Preparation of insulin-chitosan complex nanoparticles (ICCN)

Chitosan was dissolved in 1% glacial acetic acid solution, and the resulting solution's pH was adjusted to 5.8 using NaOH solution. Then, 1 ml of insulin solution (with a concentration of 6.94 mg/ml and pH 5.8) was added to the CS solution under magnetic stirring for 1 h at room temperature. Finally, DS solution with pH adjusted at 5.8 was added dropwise to the mixture of CS and insulin under stirring (CS to DS final mass ratio is to be 1.5:1), and ICCN was collected using centrifugation [18].

### (B) Preparation of insulin-loaded chitosan nanoparticles (ICN)

Chitosan was dissolved in a 1% glacial acetic acid solution. The pH value of the solution was confirmed to be 4. Then, 1 ml of insulin solution (with a concentration of 6.94 mg/ml and pH 4) was mixed with the DS solution; its pH was adjusted to 4. This mixture was then added to the CS solution under stirring. After that, the prepared nano-formulations were centrifuged and washed with deionized water.

### Silica coating of the prepared formulations

By adding NH4OH to prepared formulations, silica coating was provided to adjust their pH to 10. Then a solution of TEOS (15 µl) and ethanol (0.5 ml) was then slowly added at a rate of 10µ/min under sonication at 40 °C [19]. The Silica-coated NPs collected via centrifugation.

### Characterization of the prepared nano-formulations

The size and morphology of the prepared formulations (uncoated and silica-coated CS-DS NPs) were obtained using TEM (JEM 1230 electron microscope Jeol, Tokyo, Japan). A drop of each diluted formulation was applied to a copper grid and left to dry for 15 min. The two samples were negatively stained using 1% phosphotungstic acid and left to dry. Then, the images were captured with a high-resolution transmission electron microscope TEM. The surface structure and quantitative chemical analysis of the uncoated and silica-coated CS-DS NPs were determined using SEM (Quanta TM 250 FEG, FEI; USA), equipped with an Energy-Dispersive X-ray (EDX) spectrometer to measure the elemental concentrations in the formulations. The accelerating voltage was 20 kV. The hydrodynamic size distribution and zeta potential of nano-formulations (uncoated and silica-coated CS-DS NPs) were determined using Zetasizer (Nano ZS90, Malvern Instruments, UK) at 25 °C. The formulations were appropriately diluted with deionized water for the analysis. The Dynamic Light Scattering (DLS) technique measured the hydrodynamic size. And the zeta potential was directly measured by the migration of particles in an electric field [20]. Each measurement was analyzed three times to calculate the mean values and the standard errors. The prepared formulations' topographic properties were investigated by well-resolved non-contact AFM (Wet – SPM9600, Shimadzu, Japan). In non-contact AFM, the tip mounted on the end of a cantilever for sample surface scanning never contacts the sample leaving a space on the order of tens to hundreds of angstroms away from it and, therefore, cannot disturb or destroy the sample. The surface topography in non-contact AFM mode is measured by utilizing the attractive atomic force in the distance between the tip and a sample surface.

### Differential Scanning Calorimetry (DSC)

The Differential Scanning Calorimeter DSC131 Evo (SETARAM Inc., France) was used to evaluate the thermal behavior of the two prepared formulations (uncoated and silica-coated CS-DS NPs). The DSC analysis was performed at a heating range from 25 °C to 350 °C with a heating rate of 10 °C / min. The samples were weighed in an Aluminum crucible 120 ul and presented to the DSC. The results of the thermo-gram were processed using (CALISTO Data processing software v.149).

### Fourier Transform Infrared (FTIR) spectroscopy

The FTIR spectrometer (Edwards High Vaccum, Craeley Sussex, England) was used to assess (for the pellet of uncoated and silica-coated CS-DS NPs) the interaction between the silica coat and the surface of CS-DS NPs. FTIR scanning speed was 2 mm/sec, and the resolution was 4 cm^−1^. The resulting different spectra patterns are recorded as a relation between wave number and transmittance %

### Insulin encapsulation efficiency using HPLC

The two prepared formulations (ICCN and ICN) were centrifuged (VS-18000 M, Korea, power 220 V/ 50 HZ) at 10,000 RPM for 20 min. The supernatant of the two formulations was then collected to estimate the insulin concentration (the free drug) using the high-performance liquid chromatography technique (HPLC) (Young Lin Instrument, Korea). The flow rate was maintained at 1.0 ml/min, and the injection volume was 20 μL. The drug (insulin) was monitored by its UV absorbance at 270 nm. The run time was 9 min, and the insulin had a retention time of ~ 6 min. The insulin encapsulation efficiency was calculated for each formulation from the following equation:

$$\mathrm{Encapsulation Efficiency \%}=\frac{\mathrm{Initial concentration}-\mathrm{Supernatant concentration}}{\mathrm{Initial concentration}}\times 100\mathrm{ \%}$$

### In vitro drug release

The insulin release profiles from the prepared formulations (ICCN and ICN) were performed using the dialysis bag method (MWCO 12,000 g/mole; Sigma-Aldrich) in two different pHs (5.5, 7) to ensure the stability and controlled release of the insulin within media like those of human GIT [16]. A volume of 20 ml of phosphate buffer saline (pH 7) was prepared at 37^o^ C and introduced to each falcon tube for each formulation as a ready state for the in vitro release experiment. Equivalent volumes of each formulation (5 ml) were filled in the dialysis bags and immersed in 20 ml buffer (pH 7). The same steps were repeated for the buffer (pH 5.5). At a discreet time interval, a sample of 2 ml was withdrawn from each buffer solution (to measure the amount of insulin released) and replaced with fresh buffers. The absorbance of the samples was then measured using a UV–Vis spectrophotometer at a wavelength of 270 nm. And the concentrations of released insulin were calculated.

### Statistical analysis

DLS, zeta potential, and drug release data were measured as three replicates. The results were expressed as mean ± S.E.M. Statistical Package for Social Sciences origin software (version 94E) used for all data. Data analysis was implemented on a compatible computer.

## Results

### Physical characterization of the prepared formulations

**Fig. 1:**
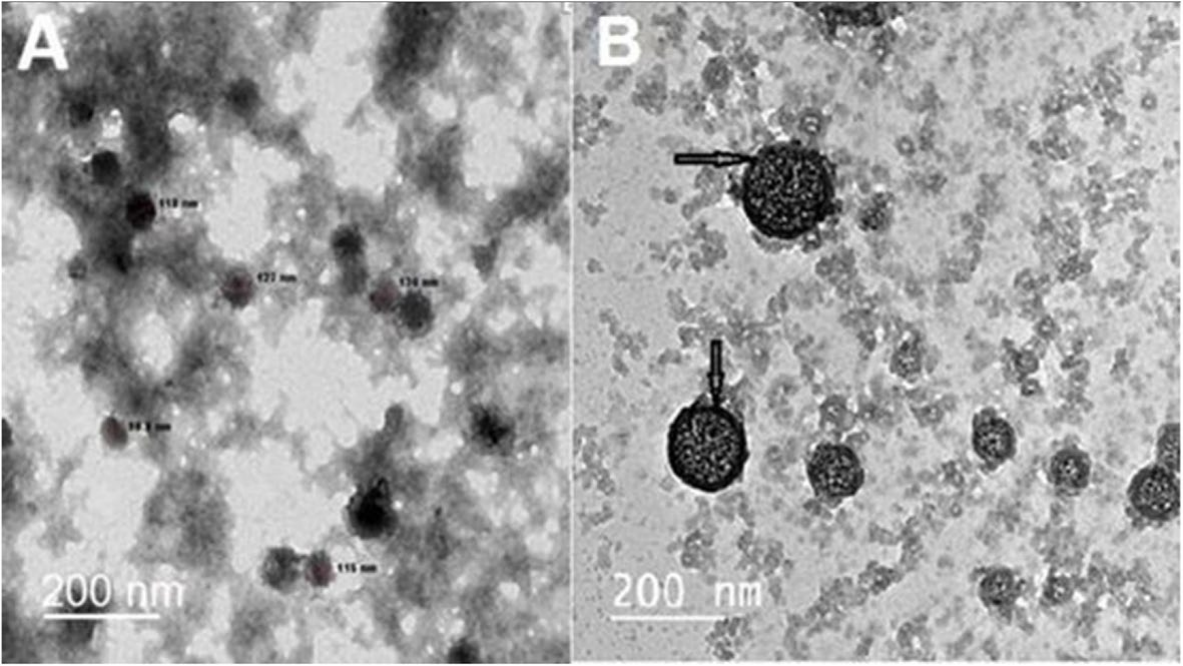
TEM images for (**A**) uncoated CS-DS NPs and (**B**) silica-coated CS-DS NPs

**Fig. 2:**
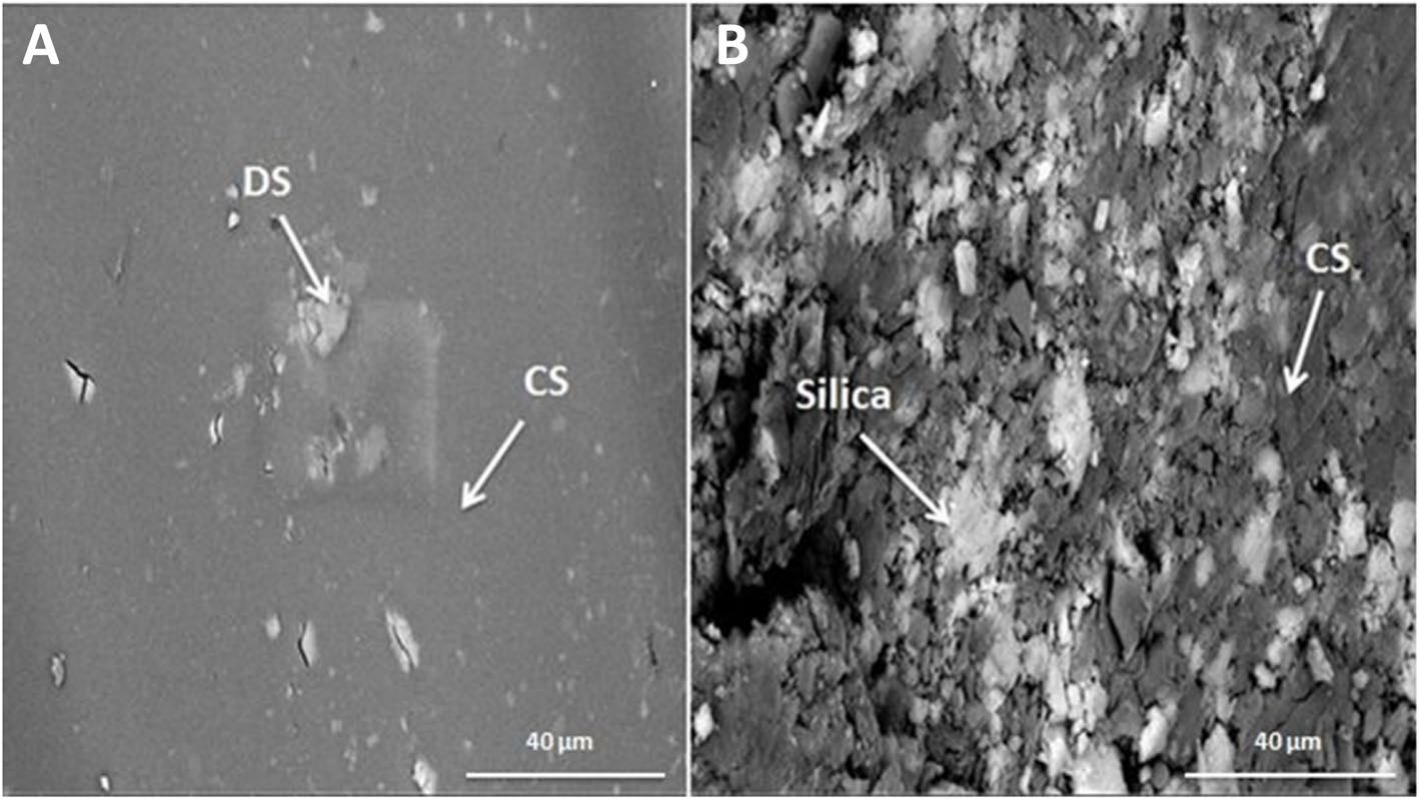
SEM images for (**A**) uncoated CS-DS NPs and (**B**) silica-coated CS-DS NPs

**Fig. 3:**
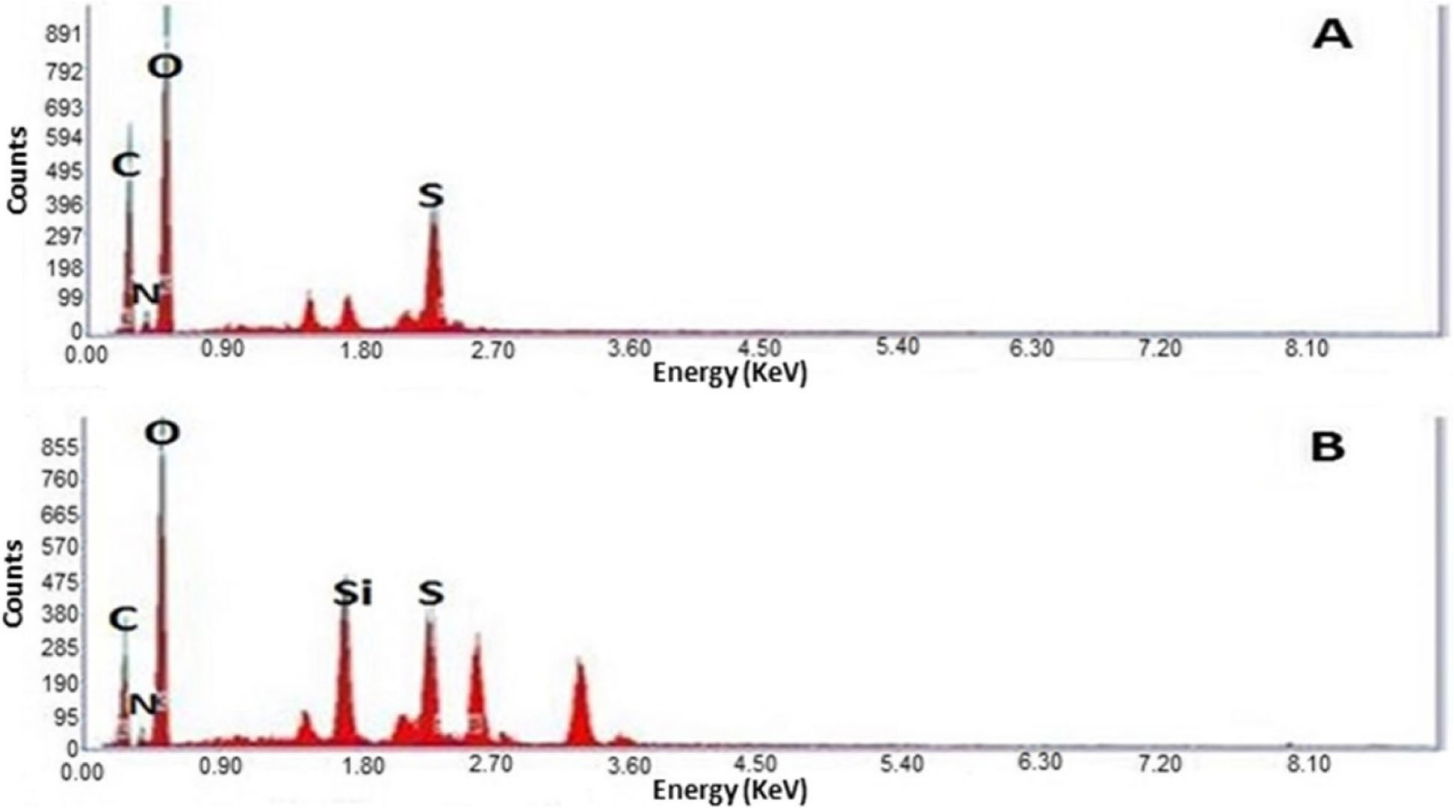
EDX spectra for (**A**) uncoated CS-DS NPs and (**B**) silica-coated CS-DS NPs

**Fig. 4:**
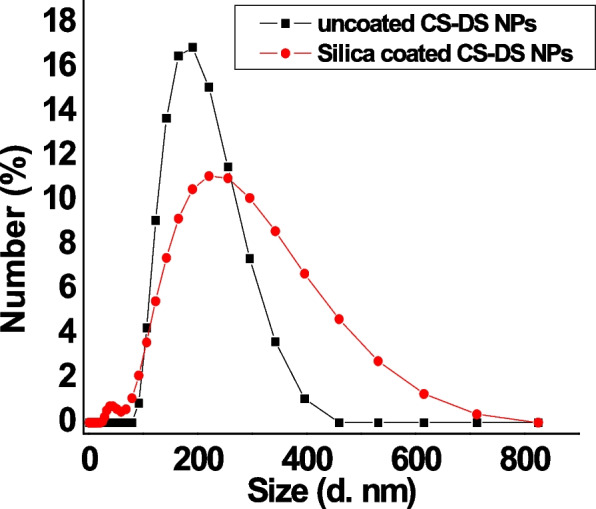
Particle size distribution for uncoated and silica-coated CS-DS NPs

**Fig. 5:**
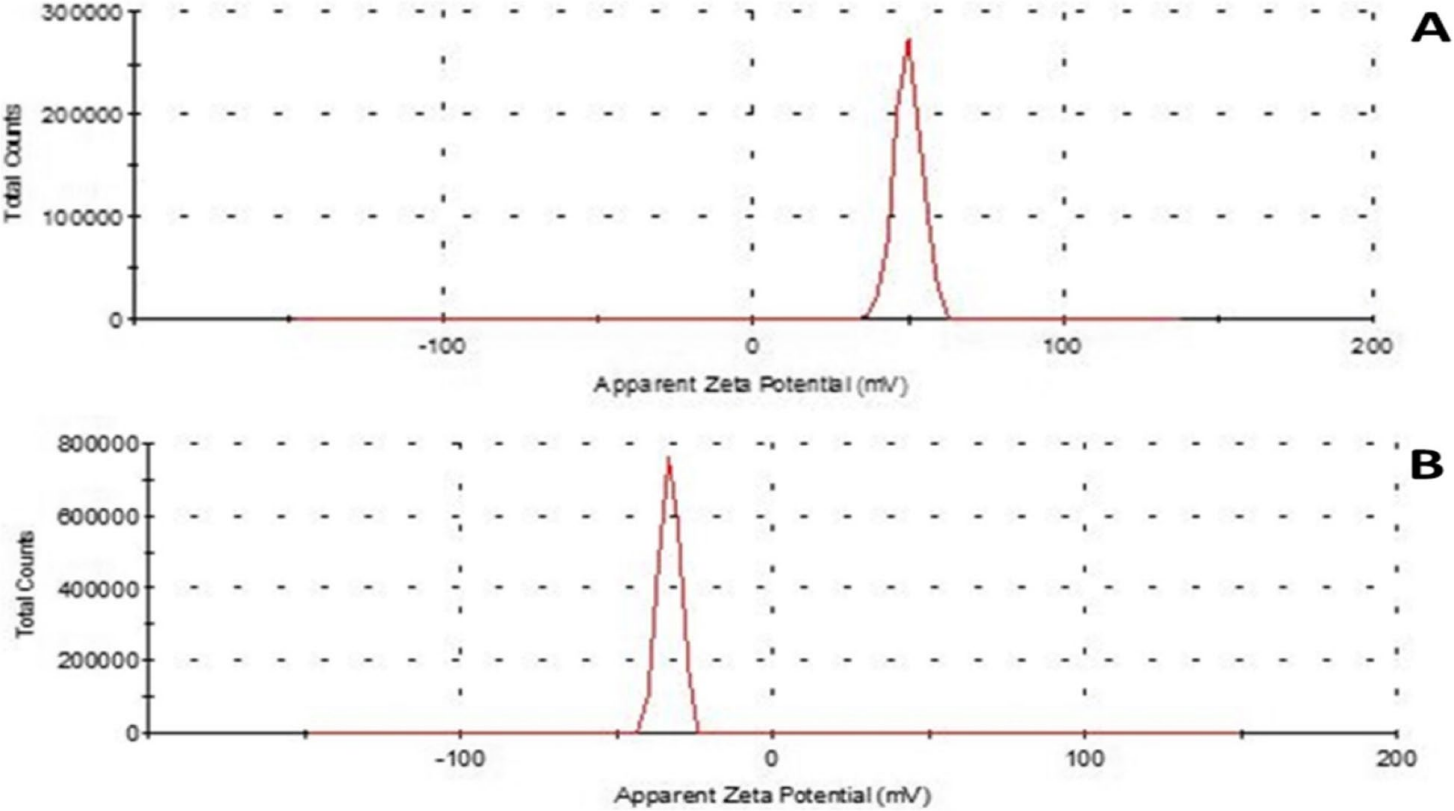
Zeta potential analysis for (**A**) uncoated CS-DS NPs and (**B**) silica-coated CS-DS NPs

**Fig. 6:**
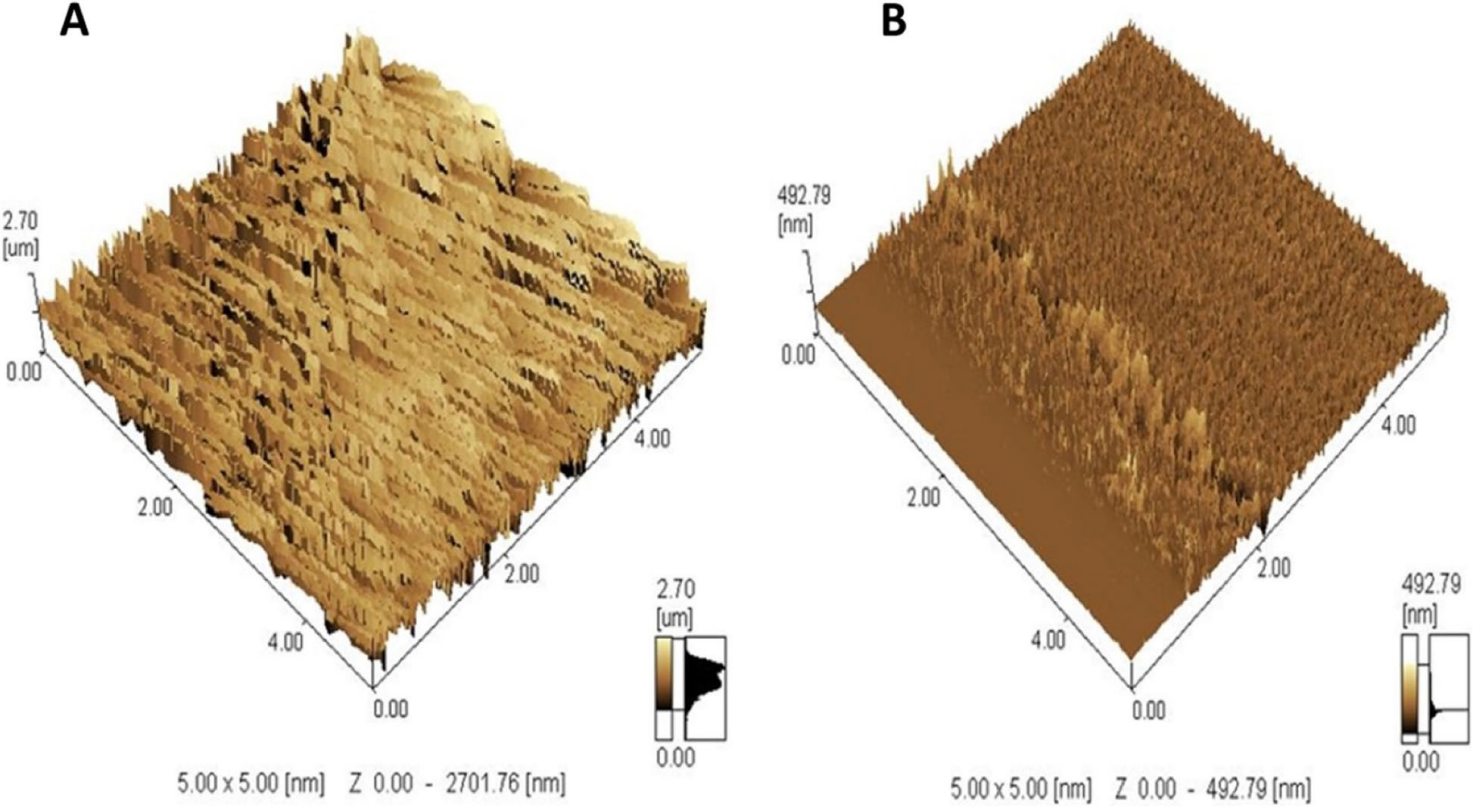
AFM images for (**A**) uncoated CS-DS NPs and (**B**) silica-coated CS-DS NPs

Figure 1 (A & B) demonstrates transmission electron micrographs for uncoated and silica-coated CS-DS NPs. TEM images showed the successful preparation of uncoated and silica-coated CS-DS NPs with homogenous sizes. The dominant morphology of the resulting particles of the two formulations was almost spherical, with few aggregations. TEM image (Fig. 1B) revealed the dense layer covered with CS-DS NPs that proved the successful formation of silica coat (arrow indicated). The resulting average nano-size of the two prepared formulations was 117 nm for uncoated CS-DS NPs and 145 nm for silica-coated CS-DS NPs. Figure. 2 shows SEM images for uncoated and silica-coated CS-DS NPs. SEM images revealed a near-coherent structure for uncoated CS-DS NPs where the nature of CS dominates the surface. In contrast, silica-coated CS-DS NPs showed a compact surface incorporating two clear phases and a porous structure. The prominent elements composing the prepared uncoated and silica-coated CS-DS NPs were determined through the EDX spectra shown in Fig. 3 (A & B). The silica (Si) absorption peaks at 1.8 keV, as seen in Fig. 3 (B). The hydrodynamic size distribution results showed that the average hydrodynamic size of silica-coated CS-DS NPs was about 210 ± 21 nm, which is larger than that of uncoated CS-DS NPs (190 ± 19 nm) (Fig. 4). The average zeta potential value of uncoated CS-DS NPs was 48.75 ± 4.88 mV, whereas that of silica-coated CS-DS NPs was -32 ± 3.2 mV (Fig. 5). The topographic images obtained by AFM for uncoated and silica-coated CS-DS NPs are demonstrated in Fig. 6 A & B, respectively. The nanoscale features of the two formulations can be observed, portraying the roughness of their materials. The average roughness values of uncoated CS-DS NPs were (7.72 ± 2.75) larger than that of silica-coated CS-DS NPs (2.21 ± 0.81).

Differential Scanning Calorimetry (DSC).

**Fig. 7:**
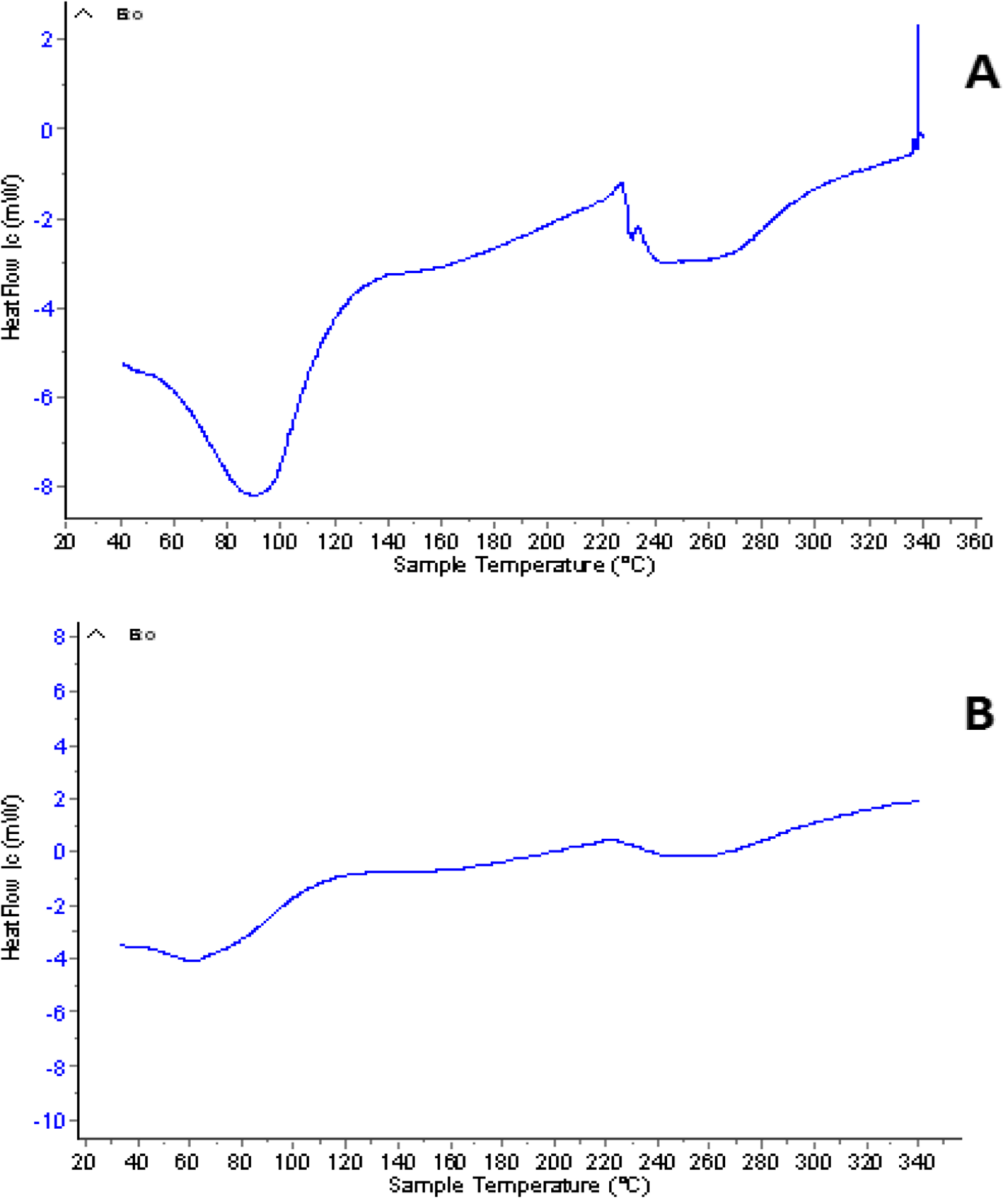
DSC of (**A**) uncoated CS-DS NPs and (**B**) silica-coated CS-DS NPs

As seen in Fig. 7A, the DSC thermogram of uncoated CS-DS NPs showed two endothermic peaks, the first at 90.747 ºC and the second at 254.5ºC, in addition to an exothermic peak at 230.8ºC. While the DSC thermogram of silica-coated CS-DS NPs (Fig. 7B) showed a shift in the first endothermic peak to a lower temperature at 64.772ºC, whereas the second endothermic peak was shifted to a higher temperature at 257.324 ºC, and the exothermic peak disappeared.

### Fourier transform infrared (FT-IR) spectroscopy

**Fig. 8:**
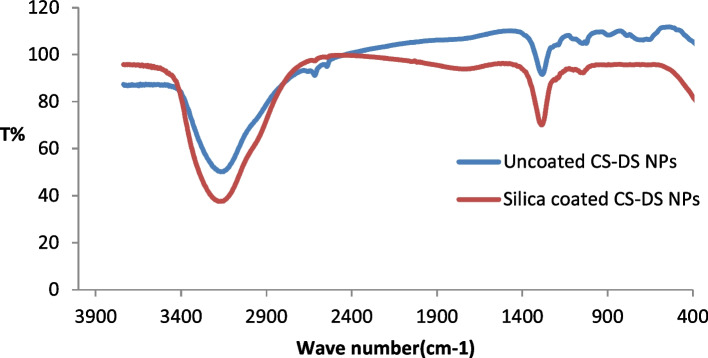
FTIR of uncoated CS-DS NPs and silica-coated CS-DS NPs

FTIR spectra of uncoated and silica-coated CS-DS NPs are shown in Fig. 8. For the uncoated CS-DS NPs spectrum, a peak corresponding to asymmetric stretching of S˭O was found at around 1233 cm^−1^[22]. An intense peak was observed at about 3114 cm^−1^, characteristic of O–H stretching and intramolecular hydrogen bonds[23]. Two tiny adjacent bands appeared in the spectrum of polysaccharides and were noticed at 2490 and 2564 cm^−1^, corresponding to the C-H symmetric and C-H asymmetric stretching, respectively, as well as small adjacent peaks were recorded at around 637, 848, and 1001 which are corresponding to SO_4_ asymmetric bending, S–O–S vibration and C-O stretching, respectively[22, 24, 25]. The FTIR spectrum of silica-coated CS-DS NPs is nearly similar to that of uncoated, but it's observed that the minor peaks at 637, 848, 2490, and 2564 almost disappeared. So it was suggested that these groups were engaged in the interaction with the silica coat.

### HPLC assay of insulin (Drug encapsulation efficiency)

The drug encapsulation efficiency of ICN prepared at pH 4 was 66.5%, whereas the encapsulation efficiency of ICCN prepared at pH 5.8 was 60.177%.

### In vitro drug release

**Fig. 9:**
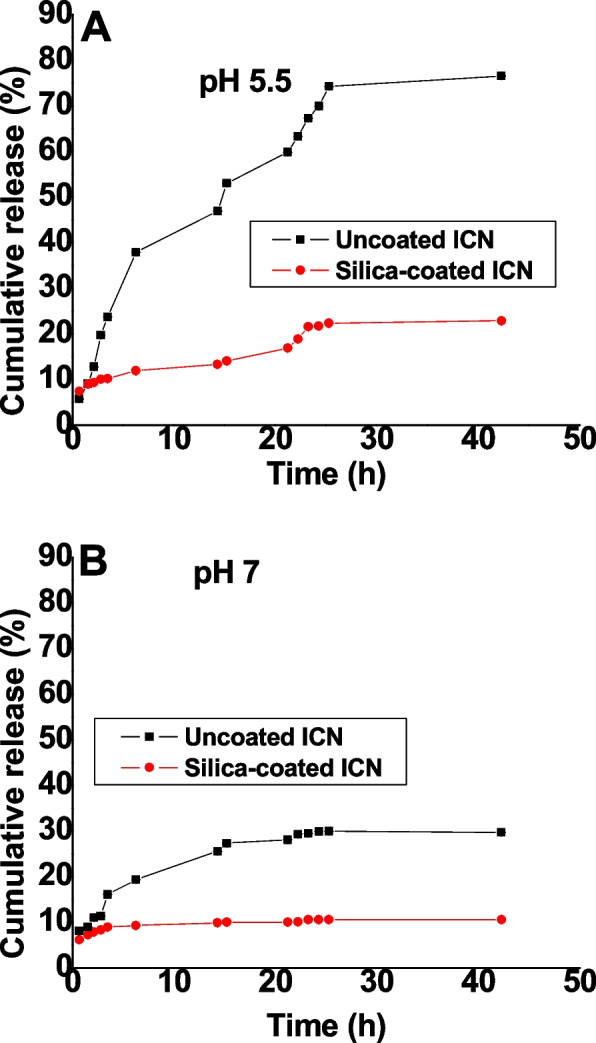
The in vitro release study of insulin from uncoated and silica-coated ICN in (A) pH 5.5 and (B) pH 7

The in-vitro release profile of insulin from uncoated and silica-coated ICN was studied to evaluate their efficiency as insulin nano-carrier. The release of insulin from uncoated and silica coated ICN at 5.5 and 7 pH were expressed in terms of cumulative release (%) versus time, as shown in Fig. 9 A&B. For acidic pH, the release behavior of insulin from uncoated ICN showed a burst release state; approximately 40% of insulin was released through the first 5 h at a relatively rapid rate, and most of the insulin amount was released within 24 h. In contrast, the release behavior of insulin from silica-coated ICN showed a slow and sustained release state. For pH 7, the insulin release from uncoated ICN was higher than that from silica-coated ICN. Only about 10% of insulin was slowly released from silica-coated ICN during the experiment.

## Discussion

To overcome insulin's oral drug delivery problems and improve the properties of CS-DS NPs as an efficient insulin carrier, this study focused on evaluating silica as a coat for CS-DS NPs and studying the emerging properties of adding the silica coat. Comprehensive analyses were executed to show the impact of the silica coat on CS-DS NPs and investigate the validity of the prepared formulation for further applications. Different physical techniques studied the properties of silica-coated CS-DS NPs compared to those of uncoated CS-DS NPs. Morphological characterization demonstrated by TEM, SEM, and AFM images confirmed a uniform layer of silica coating the surface of CS-DS NPs. The larger size of silica-coated CS-DS NPs, compared to the size of uncoated CS-DS NPs, was inferred by TEM and DLS results.

The colloidal stability of silica-coated CS-DS NPs was studied using the zeta potential technique, which is considered a fundamental analysis to evaluate the colloidal stability of nanoparticles [26]. The required minimum value for the stability assurance of a colloidal suspension system was proposed to be ± 30 mV [27, 28] as nanoparticles show poor colloidal stability at low positive or negative zeta potential and tend to agglomeration. Therefore, the measured zeta potential value of silica-coated CS-DS NPs (-32 ± 3.2 mV) indicates higher colloidal stability than uncoated CS-DS NPs having a zeta potential value of 48.75 ± 4.88 mV [29, 30]. In addition, it was proved that the ability of the negatively charged NPs to penetrate the mucus layer (coverage of the epithelial cells of the GIT) with less interaction than that emerges from the high positive NPs (characterized by a mucoadhesive property) [31]. Therefore, silica-coated CS-DS NPs are not trapped in the mucus network and can deliver the loaded drug to the underlying desired tissues. Moreover, the negative surface potential of silica-coated NPs minimizes their tendency to adsorb plasma proteins, increasing their stability in blood circulation [32].

It was found that there is an actual correlation between the surface roughness of NPs and their cellular attachment [33, 34]. Rough surfaces have a high ability for the entrapment of glycosylated proteins (mucins) composing the mucus layer lining the GIT, affecting their permeability through the epithelial cells and hindering their accessibility to blood circulation [35, 36]. Therefore, the interaction between highly rough surfaces of uncoated NPs and mucus cells decreases their in vivo stability. Hence, the lower roughness value (2.21 ± 0.81) of silica-coated CS-DS NPs, indicated by AFM, confirmed their higher bioavailability than uncoated nanoparticles with a roughness value of 7.72 ± 2.75.

For the DSC thermogram of silica-coated CS-DS NPs, the shift in the first endothermic peak to lower temperature indicated the decreased crystallinity of CS due to its entrapment with silica in addition to the interaction between polymers and silica, causing dehydration associated with hydrophilic groups of polymers [37–39]. The second endothermic peak (of CS-DS NPs) was slightly shifted to a higher temperature when adding silica which can be explained by higher formulation stability due to the formed physical crosslinking [40]. It was also noted that the intensity of endothermic peaks was decreased, which may suggest the reduced reorganization ability of the CS-DS matrix by adding silica, causing a stiffer matrix [40]. At the same time, the disappearance of the exothermic peak by adding silica may be evidence that no degradation of polymers has occurred [39]. FTIR also characterized the silica coating, whereas the disappearance of the peaks at 637 cm^−1^ and the small peaks at 2490 and 2564 cm^−1^, which correspond to the skeletal vibrations of polysaccharides, as well as the disappearance of the peak at848cm^−1^corresponding to sulfated polysaccharides and the formation of glycosidic linkages as a result of the interaction of silica coating [41]. The calculated encapsulation efficiency was higher for the lower pH method (insulin-loaded CS NPs). That may be due to the isoelectric point of insulin (PI = 5.3), meaning that insulin in acidic media less than its PI, like that of the ICN method with pH 4, has positive charges. So, when it is first mixed with negatively charged DS before adding to CS, it is expected that an electrostatic attraction occurs between the two oppositely charged molecules in addition to the electrostatic attraction that may then emerge between the positively charged CS and DS molecules resulting in high encapsulation efficiency [15, 42]. The release profiles of ICN and silica coated ICN at two different pHs simulating the media of the human GIT were assessed. The slow and sustained release was obtained in the case of silica-coated ICN at pHs (5.5, 7), The low release of silica-coated ICN is due to the silica coat, and two mechanisms suggested this: First, It was proved that the silica coating could improve the chitosan resistance against pH denaturation [43]. Second, the presence of a silica coat decreases the surface porosity of the prepared nano-carrier (demonstrated by the decreasing surface roughness values). That reduces the insulin molecules to be released, suggesting that the coating of ICN provides a protective shield allowing controlled release of the drug during its journey through pH varieties of GIT.

## Conclusion

This study presents silica-coated IC as an efficient promising strategy for the oral administration of insulin. The favorable characteristics results were obtained for silica-coated CS NPs compared to uncoated CS NPs, including the relevant nano-size indicated by TEM images and DLS results and the colloidal stability inferred from zeta potential, AFM, and DSC results. The higher encapsulation efficiency was obtained at, the lower pH loading method, silica-coated ICN, achieving sustained release results for insulin at pHs (5.5,7), mimicking the media of GIT. This work demonstrates inclusive investigation of a potential insulin delivery system as a novel challenge overcoming the main problems of oral peptides and proteins administration. It should be considered a pave the way for further research supporting its possible medical applications in biological systems.

## Data Availability

All data needed to support the conclusions are included in this article. Additional data related to this paper can be requested from the author (hfahmy@sci.cu.edu.eg).

## Abbreviations

CS: Chitosan
DS: Dextran sulfate
NPs: Nanoparticles
TEOS: Tetraethyl orthosilicate
NaOH: Sodium hydroxide
NH4OH: Ammonium hydroxide
HCL: Hydrochloric acid
ICCN: Insulin-Chitosan Complex Nanoparticles
ICN: Insulin-loaded Chitosan Nanoparticles
ml: Millieliter
mg: Millie gram
w/v: Weight per Volume
µl: Microliter
IU: International unit
C: Degree Celsius
RPM: Revolutions per minute
TEM: Transmission electron microscopy
SEM: Scanning electron microscopy
EDX: Energy-Dispersive X-ray
DLS: Dynamic Light Scattering
AFM: Atomic Force Microscopy
DSC: Differential Scanning Calorimetry
FTIR: Fourier transform infrared spectroscopy
S.M.E.: Standard error of the mean
HPLC: High-Performance Liquid Chromatography technique
UV–Vis: Ultraviolet–visible
GIT: Gastrointestinal tract
PI: Isoelectric point of insulin
